# A Longitudinal Evaluation of an Intervention Program for Physical Education Teachers to Promote Adolescent Motivation and Physical Activity in Leisure Time: A Study Protocol

**DOI:** 10.3390/mps8020034

**Published:** 2025-04-01

**Authors:** Hasso Paap, Andre Koka, Henri Tilga

**Affiliations:** Institute of Sport Sciences and Physiotherapy, University of Tartu, Ujula 4, 51008 Tartu, Estonia; andre.koka@ut.ee (A.K.); henri.tilga@ut.ee (H.T.)

**Keywords:** children, adolescents, self-determination theory, autonomy, competence, relatedness, motivation, physical education, physical activity, intervention

## Abstract

(1) Background: Research has consistently demonstrated that regular physical activity (PA) is associated with several benefits among adolescents. However, PA levels among adolescents are low worldwide and tend to decrease with age. Consequently, researchers aim to identify psychological antecedents of PA to inform effective interventions, including in physical education (PE) settings. PE lessons provide an ideal environment for conveying health-related messages to adolescents. (2) Methods: This project aims to develop a three-month face-to-face and web-based intervention program for PE teachers to increase autonomy-, competence-, and relatedness-supportive behavior toward their students (main trial phase 1: min *n* = 78; main trial phase 2: min *n* = 116) and to avoid the respective need-thwarting behaviors toward their students. The effectiveness of the intervention program is examined at multiple time points during the three-month period. (3) Results: After the intervention program, it is expected that the experimental group students demonstrate significantly higher intrinsic motivation toward PA and are significantly more physically active, as measured by accelerometers, compared to control group students. Additionally, the unique effects of autonomy, competence, and relatedness support interventions on students’ intrinsic motivation and PA will be investigated. (4) Conclusions: This project provides highly valuable insights for PE teacher training to increase students’ intrinsic motivation and their overall PA.

## 1. Introduction

Research has consistently demonstrated that regular physical activity (PA) is related to several cognitive, psychological, mental, and physical benefits among adolescents [1]. However, PA levels among adolescents are low worldwide [2] and tend to decrease with age [3]. Low PA during childhood and adolescence increases the risk of chronic diseases in adulthood, leading to higher morbidity and lower quality of life [4]. As a result, researchers aim to identify psychological antecedents of PA to inform effective interventions.

Physical education (PE) lessons provide a great environment for conveying health-related messages to adolescents [5]. PE teachers can support students’ autonomy, competence, and relatedness, which, in turn, fosters intrinsic motivation in both PE and leisure-time PA. The trans-contextual model of motivation (TCM) has been extensively used to identify PA determinants in these contexts [6]. This model integrates self-determination theory (SDT) [7], the theory of planned behavior [8], and Vallerand’s hierarchical model of motivation [9]. One core assumption is that psychological experiences in one context (e.g., PE) influence others (e.g., leisure time) [6].

To further enrich our understanding of the national PE model, it is crucial to explore how psychological antecedents of PA can be effectively integrated into PE settings. Recent findings from the 2022 Estonian Physical Activity Report Card [10] reveal that despite high participation in organized sports, many adolescents do not meet recommended PA levels, indicating a need for targeted interventions. Furthermore, insights from the Global Matrix 4.0 [11] highlight global trends in PA among adolescents, which can inform our approach to enhancing PE practices. By focusing on fostering autonomy, competence, and relatedness in PE, we aim to address the identified gaps and promote intrinsic motivation for PA among students.

Studies indicate that PE teachers significantly enhance students’ intrinsic motivation [12,13]. Enhancing PE teachers’ autonomy-supportive behaviors is critical [14]. Recent research shows that combining web-based and face-to-face interventions effectively improves PE-related outcomes, such as basic psychological need (BPN) satisfaction and autonomous forms of motivation [15,16].

Few TCM-guided studies have aimed to change students’ PA behavior during leisure time (e.g., [17,18,19]), and previous autonomy-supportive interventions have been only partially effective (e.g., [20,21,22,23]). There is a scarcity of intervention studies supporting an SDT-based multidimensional approach to promote adolescents’ PA participation [24]. Recent findings emphasize the importance of providing autonomy, competence, and relatedness support to fully satisfy BPN-s and enhance intrinsic motivation [25]. It is also crucial to avoid need-thwarting behaviors, as these negatively impact adolescents’ outcomes [25,26,27,28].

In a Delphi study by Ahmadi and colleagues [25], a classification system of autonomy-, competence-, and relatedness-supportive and -thwarting behaviors was developed, resulting in a taxonomy of 57 teacher motivational behaviors, with examples including “allowing students to progress at their own pace” and “teaching students to set intrinsic life goals for learning”. A shortlist of 22 behaviors was created of those behaviors with mean score effect ratings greater than +2 or less than −2, categorized as need-supportive and need-thwarting teaching behaviors, respectively. This shortlist includes behaviors such as “praising improvement or effort” and “chaotic or absent teaching”. This classification by Ahmadi et al. [25] is the first to systematically compile expert perspectives on motivational teacher behaviors in educational settings. Building on this taxonomy, researchers can test and apply these behaviors in the school context. Recently, Ferriz and colleagues [29] adapted these behaviors to the PE context. Their project builds on these classification systems and aims to develop and test an intervention program to enhance PE teachers’ supportive behaviors and reduce their thwarting behaviors toward students.

To our best knowledge, no studies have combined face-to-face and web-based interventions in PE to enhance teachers’ need-supportive behaviors while diminishing need-thwarting behaviors, with the aim of increasing students’ intrinsic motivation in PE and leisure-time PA. The main objective of this study is to develop and test the main and combined effects of the PE teachers’ face-to-face and web-based need-supportive intervention on grade 6 to 7 adolescents’ leisure-time PA. The secondary objective of the project is to test the unique effects of interventions focused on support for each psychological need (i.e., autonomy, competence, and relatedness) separately.

## 2. Materials and Methods

### 2.1. Participants

This longitudinal intervention study will include eligible participants who are qualified full-time secondary school PE teachers and their 6th-to-7th-grade students, without restrictions on their participation in PE classes. It is important to note that the inclusion criteria for PE teachers do not specify previous work experience or the nature and extent of prior training; the key requirement is that they are full-time educators. Students who do not participate in PE classes for health reasons are not eligible to participate in this study. For the grouping of participants in this study, neither the prior knowledge of the PE teachers nor the students’ motivational levels or other examined factors will be considered; instead, groups will be formed based on random assignment, which may lead to differing baseline levels among the participants. Baseline differences in study characteristics will be assessed to clarify the effectiveness of group allocation.

For main trial phase 1, the statistical power analysis revealed that a minimum sample size of 78 students (i.e., *n* = 39 students allocated to each of the two study groups) would be required to achieve 80% statistical power to detect medium effect sizes when considering three measurements (baseline vs. one-month vs. three-month follow-up) at the within-subject level (correlated at 0.50), with an alpha level of 0.05. However, considering the possible dropout rate of approximately 40% evident in multiple follow-up studies of physical activity [30], the investigators aim to recruit 130 students at baseline (i.e., 65 students allocated to each of the two study groups).

For main trial phase 2, the statistical power analysis revealed that a minimum sample size of 116 students (i.e., *n* = 29 students allocated to each of the four study groups) would be required to achieve 80% statistical power to detect medium effect sizes when considering three measurements (baseline vs. one-month vs. three-month follow-up) at the within-subject level (correlated at 0.50), with an alpha level of 0.05. However, considering the possible dropout rate of approximately 40% evident in multiple follow-up studies of physical activity [30], the investigators aim to recruit 196 students at baseline (i.e., 49 students allocated to each of the four study groups).

Data from participants who drop out will not be replaced. Furthermore, the questionnaires have been designed within the REDCap environment in a manner that does not allow participants to skip questions or leave them unanswered.

### 2.2. Ethical Considerations, Consent, and Permissions

The research project will be conducted in accordance with the Declaration of Helsinki [31] and the guidelines of Good Clinical Practice [32]. Ethical approval for the study was obtained from the Research Ethics Committee of the University of Tartu (code: 385/T-20). First, the authorities of randomly selected public schools will be contacted with a request for permission to conduct the study in their school. The school authorities will be presented with the study details, including the explanation that participation is voluntary, and that withdrawal does not entail any negative consequences. In case of refusal, the next public school will be contacted. After permission is obtained from the school authorities, the invitation including the study information and informed consent form will be sent to the PE teachers at the school. After consent is obtained from PE teachers, the study information and informed consent forms will be distributed to the parents of all eligible students.

The study information for the participants contains an explanation of the aim and procedures of the study, the expected duration, and potential benefits and risks. The research does not harm the subjects mentally nor physically. Invasive research methods will not be used. The participants will be informed that participation is entirely voluntary, the data will be handled confidentially, and participants can withdraw from the study at any time without any negative consequences except forgoing the potential benefits of taking part in the intervention program. It will be affirmed that the anonymity of the participants will be guaranteed while presenting the data at conferences and publishing findings in peer-reviewed publications and in articles directed to the wider public.

As responses to questionnaires are confidential and anonymous, a personal code is created for each participant, to match the responses to questionnaires in various data points. The personal code for students will be based on the following characteristics: the first two letters of the first names of both parents, the name of the school, the participant’s gender, age, and the first two letters of their place of residence (city, town, or village).

The code constructed using the aforementioned information is crucial for conducting inter-group comparisons (for instance, categorizing by gender and age) and for linking questionnaires from different phases of the study during follow-up research.

The consent forms will be distributed to participants by research team members who will provide detailed information about the study.

### 2.3. Interventions

**Figure 1:**
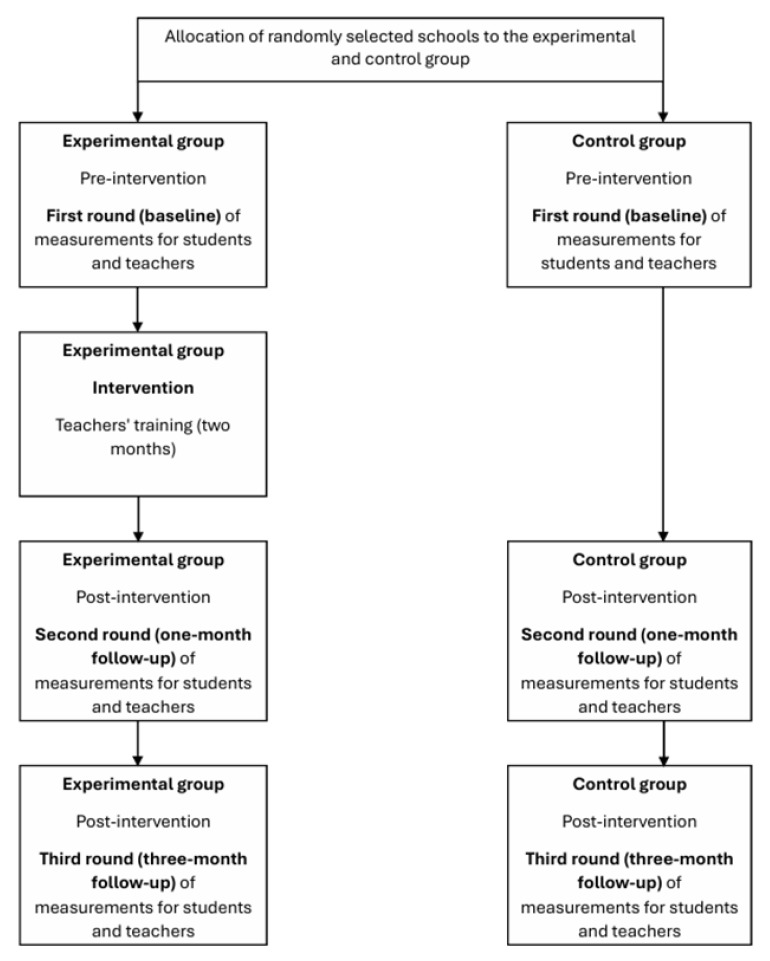
Main trial phase 1 study design.

For main trial phase 1 (Figure 1), there will be a combined face-to-face and web-based autonomy, competence, and relatedness need-supportive and need-thwarting intervention program with a duration of two months (teachers’ training). During these two months, there will be three face-to-face meetings: at the beginning of the course, at the beginning of the fifth week of the course, and at the beginning of the eighth week of the course (each lasting approximately four hours and consisting of lectures and practical activities with discussions).

Additionally, there will be new materials for PE teachers provided weekly via a web-based learning platform (Moodle) developed for this project. PE teachers will be required to report weekly on how they provide need support to their students and avoid need-thwarting behaviors through the web-based learning platform. Throughout this online course, PE teachers will receive specific materials about all need-supportive behaviors (a total of 9 specific behaviors) and need-thwarting behaviors (a total of 13 specific behaviors) proposed by Ahmadi and colleagues [25], and adapted for PE by Ferriz and colleagues [29]. This material contains detailed descriptions of each behavior along with several real-life examples. Additionally, PE teachers will complete a topic-related test at the end of each week in an online environment to demonstrate their understanding of the material. It is important to note that throughout the intervention program and training, all teachers in the intervention group will receive support to fulfill these outlined tasks. However, if any teacher fails to meet these requirements, their results, as well as those of their participating students, will not be considered. This is because, in such cases, we cannot be certain that the teacher has genuinely acquired the necessary knowledge during the training.

**Figure 2:**
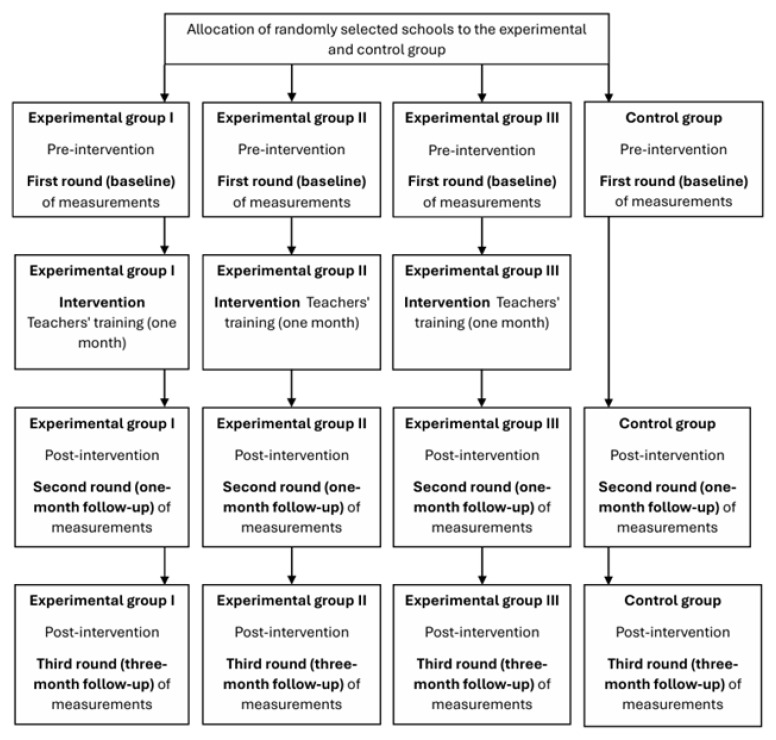
Main trial phase 2 study design.

For main trial phase 2 (Figure 2), there will be three different courses, and consequently, there will be three distinct experimental groups: (I) a combined face-to-face and web-based autonomy need-supportive intervention program with a duration of one month; (II) a combined face-to-face and web-based competence need-supportive intervention program with a duration of one month; and (III) a combined face-to-face and web-based relatedness need-supportive intervention program with a duration of one month. All these courses will be identical, with the exception that each course focuses on a specific basic psychological need—autonomy, competence, or relatedness. Each course will begin with a face-to-face meeting, and each course will end with a face-to-face meeting. The content of these face-to-face meetings will be similar to that of the face-to-face meeting held in main trial phase 1.

### 2.4. Measures

All measures (behavioral measures for students and psychological measures for students and teachers) in this study will be taken at baseline, one-month follow-up, and three-month follow-up.

#### 2.4.1. Behavioral Measure

The primary behavioral outcome measure is adolescents’ participation in leisure-time moderate-to-vigorous physical activity (MVPA), which will be examined by using Actigraph GT3X accelerometers. The sampling interval was set at 15 s. Accelerometer data were considered valid if over 600 min (10 h) of data were recorded per day with the presence of data for at least four days out of seven. Zero activity counts of 60 consecutive min were classified as non-wear time. The moderate-to-vigorous PA level in accelerometers was measured using recommended cut-off points (i.e., ≥2296 counts/min) developed by Evenson et al. [33]. In the current study, adolescents’ MVPA is measured due to its recognized health benefits, such as improved mental health, enhanced cardiorespiratory fitness, and reduced fat gain among adolescents [34]. This project focuses on promoting adolescents’ MVPA during their leisure time; therefore, accelerometer data collected during the school day will be excluded from the analyses. Additionally, accelerometer diary data will be utilized to identify the duration of MVPA in both in-school and out-of-school contexts.

#### 2.4.2. Psychological Measures for Students

The self-reported questionnaire includes variables from the TCM, such as self-reported (de-)motivating teaching, examined by the Situations in School Questionnaire (SIS [35]), adapted to the PE context [36]. We will also examine psychological need satisfaction and frustration in PE [26], different forms of motivation as described in [37], and different forms of motivation in the leisure-time physical activity context [38]. Additionally, we will measure attitudes, subjective norms, perceived behavioral control, and intentions for leisure-time physical activity [39], as well as self-reported PA [40]. Previous research has supported the validity and reliability of these measures in an Estonian adolescent sample [41].

#### 2.4.3. Psychological Measures for Teachers

The PE teachers’ questionnaire includes self-reported measures of (de-)motivating teaching, examined by the Situations in School Questionnaire (SIS [35]), adapted to the PE context [36].

### 2.5. Sample Size Calculation and Statistical Analysis

The sample size calculation will account for the nested structure of the data, specifically students nested within schools. While multilevel analysis is considered, estimating variance at the class level may not be feasible due to limited information. Instead, variances will be estimated at the school and student levels following established guidelines. It is expected that the intercepts will not significantly vary across schools for all study variables. Therefore, the main analyses will be conducted using single-level analysis at the student level.

## 3. Expected Results

The planned intervention programs are anticipated to produce meaningful advancements in the need-supportive teaching behaviors of PE teachers and, subsequently, enhance their students’ motivational and behavioral outcomes. The interventions, grounded in SDT, are designed to systematically address the basic psychological needs of autonomy, competence, and relatedness, aiming to cultivate need-supportive environments while mitigating need-thwarting practices.

During phase 1, the combined face-to-face and web-based intervention is expected to increase teachers’ awareness of need-supportive and need-thwarting behaviors. Exposure to detailed theoretical and practical knowledge of 9 need-supportive and 13 need-thwarting behaviors, based on the work of Ahmadi et al. [25] and Ferriz et al. [29], is expected to deepen teachers’ understanding of how their actions affect students’ psychological needs. Weekly assessments and reflective reporting are anticipated to enhance teachers’ self-regulation and reinforce learning. By engaging in structured, practice-oriented activities during face-to-face meetings and regular online reflections, PE teachers are expected to demonstrate an increased capacity to adopt autonomy-supportive, competence-enhancing, and relatedness-promoting behaviors in their instructional practices. Through increases in teachers’ awareness of need-thwarting practices that undermine students’ motivation [42], it is expected that such behaviors will decrease, promoting more inclusive and supportive learning environments. This is particularly important as controlling behaviors from significant others can hinder and frustrate psychological needs, which in turn may lead to amotivation [43]. The improved teaching practices resulting from the intervention are expected to enhance students’ psychological need satisfaction, leading to increased autonomous forms of motivation, and leisure-time PA, as supported by Barkoukis et al. [44]. However, it is important to note that our study will not conduct separate long-term measurements specifically related to these outcomes, focusing instead on immediate and short-term assessments of motivation and leisure-time PA.

Phase 2, consisting of focused one-month interventions on autonomy, competence, and relatedness, is anticipated to yield several key outcomes. Through the tailoring of each intervention to a single need, it is expected that teachers will develop a deeper, more nuanced understanding of autonomy-supportive, competence-enhancing, or relatedness-promoting practices. This specialized training is likely to improve targeted instructional behaviors. Focusing on specific needs across distinct intervention periods will enable teachers to differentiate and integrate strategies effectively, ensuring a more balanced and comprehensive approach to supporting students’ psychological needs. The sequential design of the interventions allows teachers to build on previous learnings, facilitating the generalization of targeted practices across different psychological need domains. By focusing on autonomy, competence, and relatedness sequentially, the interventions are expected to lead to long-term improvements in students’ autonomous forms of motivation and leisure-time PA.

In the study by Paap et al. [17], teachers were trained based on the framework established by Teixeira et al. [45] to better support students’ psychological basic needs. However, significant changes in students’ PA levels were not observed. This raises important considerations regarding the applicability of the frameworks used in PE contexts.

Ahmadi et al. [25] developed a classification system specifically tailored for educational settings, which may be more suitable for PE classes. In contrast to Teixeira et al. [45], who focused primarily on health-related contexts and presented techniques solely aimed at supporting needs, Ahmadi et al. [25] also address techniques that may threaten these needs. This distinction is crucial, as previous research indicates that both support of and threat to psychological needs can influence students through different motivational pathways [26].

By incorporating these perspectives, our study contributes to a more nuanced understanding of how various approaches can impact student engagement and PA, emphasizing the importance of context-specific strategies.

Potential limitations of the study design include the following: a higher dropout rate among students during the study than initially anticipated; and not all teachers participating in the intervention program meeting the established training requirements.

In conclusion, the proposed intervention programs are designed to facilitate systemic changes in PE teachers’ teaching practices, thereby fostering need-supportive educational environments. The outcomes of these interventions are expected to contribute significantly to the advancement of both teacher professional development and student well-being, aligning with broader educational and public health goals.

## Data Availability

Data from the experimental study will be available in the Open Science Framework (OSF) data repository.
